# BHC-YOLOV8 : improved YOLOv8-based BHC target detection model for tea leaf disease and defect in real-world scenarios

**DOI:** 10.3389/fpls.2024.1492504

**Published:** 2024-12-02

**Authors:** BaiShao Zhan, Xi Xiong, Xiaoli Li, Wei Luo

**Affiliations:** ^1^ School of Electrical and Automation Engineering, East China Jiaotong University, Nanchang, China; ^2^ College of Biosystems Engineering and Food Science, Zhejiang University, Hangzhou, China

**Keywords:** BiFormer, Haar, down sampling, skip connections, YOLOv8, tea

## Abstract

**Introduction:**

The detection efficiency of tea diseases and defects ensures the quality and yield of tea. However, in actual production, on the one hand, the tea plantation has high mountains and long roads, and the safety of inspection personnel cannot be guaranteed; on the other hand, the inspection personnel have factors such as lack of experience and fatigue, resulting in incomplete and slow testing results. Introducing visual inspection technology can avoid the above problems.

**Methods:**

Firstly, a dynamic sparse attention mechanism (Bi Former) is introduced into the model backbone. It filters out irrelevant key value pairs at the coarse region level, utilizing sparsity to save computation and memory; jointly apply fine region token to token attention in the remaining candidate regions. Secondly, Haar wavelets are introduced to improve the down sampling module. By processing the input information flow horizontally, vertically, and diagonally, the original image is reconstructed. Finally, a new feature fusion network is designed using a multi-head attention mechanism to decompose the main network into several cascaded stages, each stage comprising a sub-backbone for parallel processing of different features. Simultaneously, skip connections are performed on features from the same layer, and unbounded fusion weight normalization is introduced to constrain the range of each weight value.

**Results:**

After the above improvements, the confidence level of the current mainstream models increased by 7.1%, mAP0.5 increased by 8%, and reached 94.5%. After conducting ablation experiments and comparing with mainstream models, the feature fusion network proposed in this paper reduced computational complexity by 10.6 GFlops, increased confidence by 2.7%, and increased mAP0.5 by 3.2%.

**Discussion:**

This paper developed a new network based on YOLOv8 to overcome the difficulties of tea diseases and defects such as small target, multiple occlusion and complex background.

## 1 Introduction

Tea leaf defects and diseases significantly impact both the yield and quality of tea. Statistics show that these issues result in an annual loss of nearly 5% of tea production (Chen et al., 2020). Traditional preventive measures heavily rely on farmers’ experience and manual inspection, which present various challenges (Atila et al., 2021). Some tea gardens are located in steep terrains, making timely inspections difficult. Additionally, large areas of tea cultivation mean that manual inspection efficiency is low, posing potential risks (Baranwal et al., 2021). Given the current production landscape, manual identification methods are insufficient to meet the demands of modern large-scale cultivation (Barburiceanu et al., 2021).

With the continuous development of image processing technology, traditional manual agriculture in China is transitioning towards computerized, intelligent, and digital agriculture (Dhaka et al., 2021). Utilizing computer vision (Li et al., 2022) to prevent tea leaf defects not only reduces economic losses from manual labor but also enhances tea yield and quality (Tiwari et al., 2021). Sun et al. (2019) proposed a new method combining simple linear iterative cluster and SVM to achieve accurate of tea tree leaf disease salinity maps in a complex background context. With the advancement of deep learning, an increasing number of researchers are exploring its application in detecting crop leaf diseases and pest infestations. The rise of image recognition technologies has particularly highlighted the effectiveness of convolutional neural networks (CNNs) in the automatic classification and identification of plant diseases (Chen et al., 2019). For example, Chen et al. (2019) developed a CNN model named LeafNet, designed to automatically extract features from images of tea tree diseases.

While the above methods have performed well in the treatment of crop diseases, they focus solely on either crop disease image identification or classification. In recent years, with the rapid development of chip computing power, deep learning technology relying on computing power has also been applied in the field of image detection and processing. Its advantages are mainly reflected in its powerful feature extraction ability, high accuracy, strong generalization ability, real-time performance, and intelligent processing (Wang et al., 2024b). Algorithms based on deep learning can learn effective feature representations from massive image data, capturing subtle and complex features, which is crucial for accurate detection; meanwhile, deep learning models can learn advanced features of images and accurately detect and classify new, unseen images. Image detection networks based on deep learning have been categorized into two main types: two-stage and one-stage detection networks (Jiao et al., 2019). Faster Region-Based Convolutional Neural Networks (Faster R-CNN) stand out as a prominent example of the former. Although Faster R-CNN offers high detection accuracy (Ren et al., 2016), its slower processing speed fails to meet real-time application demands. In contrast, one-stage detection networks, including You Only Look Once (YOLO) (Redmon et al., 2016), Single Shot MultiBox Detector (SSD) (Liu et al., 2016), and RetinaNet (Lin et al., 2017), are favored for their efficiency. The YOLO family, in particular, has gained significant traction in agriculture due to its ability to deliver both speedy and accurate detections. Tian et al. (2019) employed YOLOv3 to design a system capable of real-time detection of apples at three different growth stages within an orchard. Roy et al. (2022) enhanced YOLOv4 to create a high-performance, real-time, fine-grained target detection framework adept at navigating challenges such as dense distribution and irregular morphology. Sun et al. (2022) introduced an innovative approach by integrating the YOLO-v4 deep learning network with computer graphics algorithms for improved segmentation of overlapping tree crowns. Additionally, Dai and Fan (2022) developed a crop leaf disease detection method named YOLOv5-CAcT, which is based on the latest YOLOv5 model, showcasing the ongoing evolution and application of these networks in agricultural settings. Weihao et al. (2023) proposed a tea disease identification model based on YOLOv7, achieving a recognition accuracy of 94.2% for five types of tea diseases. However, these methods were trained on single leaf datasets rather than directly captured from tea plants in real production environments, limiting their applicability in practical scenarios.

In production and daily life, drone inspection is a very practical means. However, in order to ensure their own safety, drones need to be 40-100cm away from tea trees, and the captured images will inevitably capture fallen leaves and weeds in the gaps between tea trees (Yuan et al., 2022), which will seriously interfere with the accuracy of the model. To solve the above problems, this paper inserts the BiFormer attention module into the backbone layer and adds a detection head to improve the detection success rate in complex backgrounds; at the same time, conventional sampling modules cannot distinguish between fallen leaves and pests and diseases. This paper introduces Haar wavelet function to improve the downsampling module, which can identify disease defects without interference from fallen leaves and weeds. Finally, in order to ensure the lightweighting of the model, a new feature fusion network was designed for the entire model to reduce computational complexity and facilitate deployment on mobile devices.

## 2 Materials and methods

### 2.1 Image data

**Figure 1:**
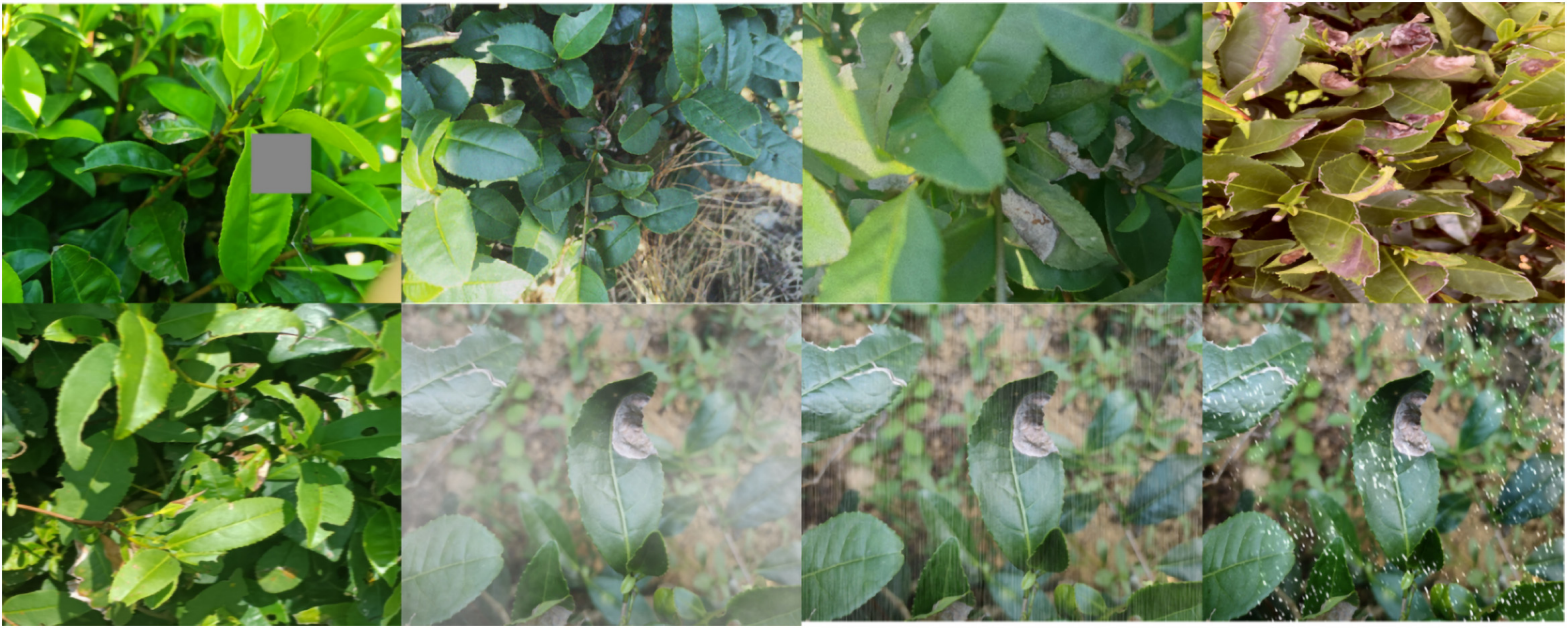
Tomato samples and cross sections.

Due to the lack of authoritative public tea datasets, the data used in this article was collected in April and May at longitude 115°8'14.54''E and latitude 32°43'47.75''N. These images were captured under natural light using a Huawei Mate60 portable device and a Sony ILME-FX30B camera, with a total of 4000 data samples collected. The pixel resolution of the image is 3024 * 4032. Among them, tea farmers and tea experts identified 43 images as red leaf spots, 213 images as algal leaf spots, 324 images as bird eye disease, 1102 images as gray wilt, 43 images as white spots, 75 images as anthrax, 1213 images as brown wilt, and 987 images as healthy leaves. Due to the limited data collected on tea defects and diseases, and the fact that the images were taken under clear weather conditions, this paper simulated adverse conditions to improve the generalization performance of the model. These simulation conditions include defocused images, partial data loss, heavy rain and snow. Data augmentation simulated conditions such as partial image loss, motion blur, early morning and dusk lighting, as well as fog, rain, snow, and wind. This method not only simulates various situations encountered in actual production, but also improves the generalization performance of the training dataset. After scaling up the original dataset by 2.5 times, a total of 10000 images were obtained. The dataset includes 10000 annotated bounding boxes (BBOX) for all defect types. Among them, 80% is the training set and 20% is the validation set. Each bounding box is manually annotated using open-source annotation tools to ensure that every defect is fully included in BBOX. **Figure 1** shows a subset of the enhanced dataset.

### 2.2 YOLOv8 detection algorithm

**Figure 2:**
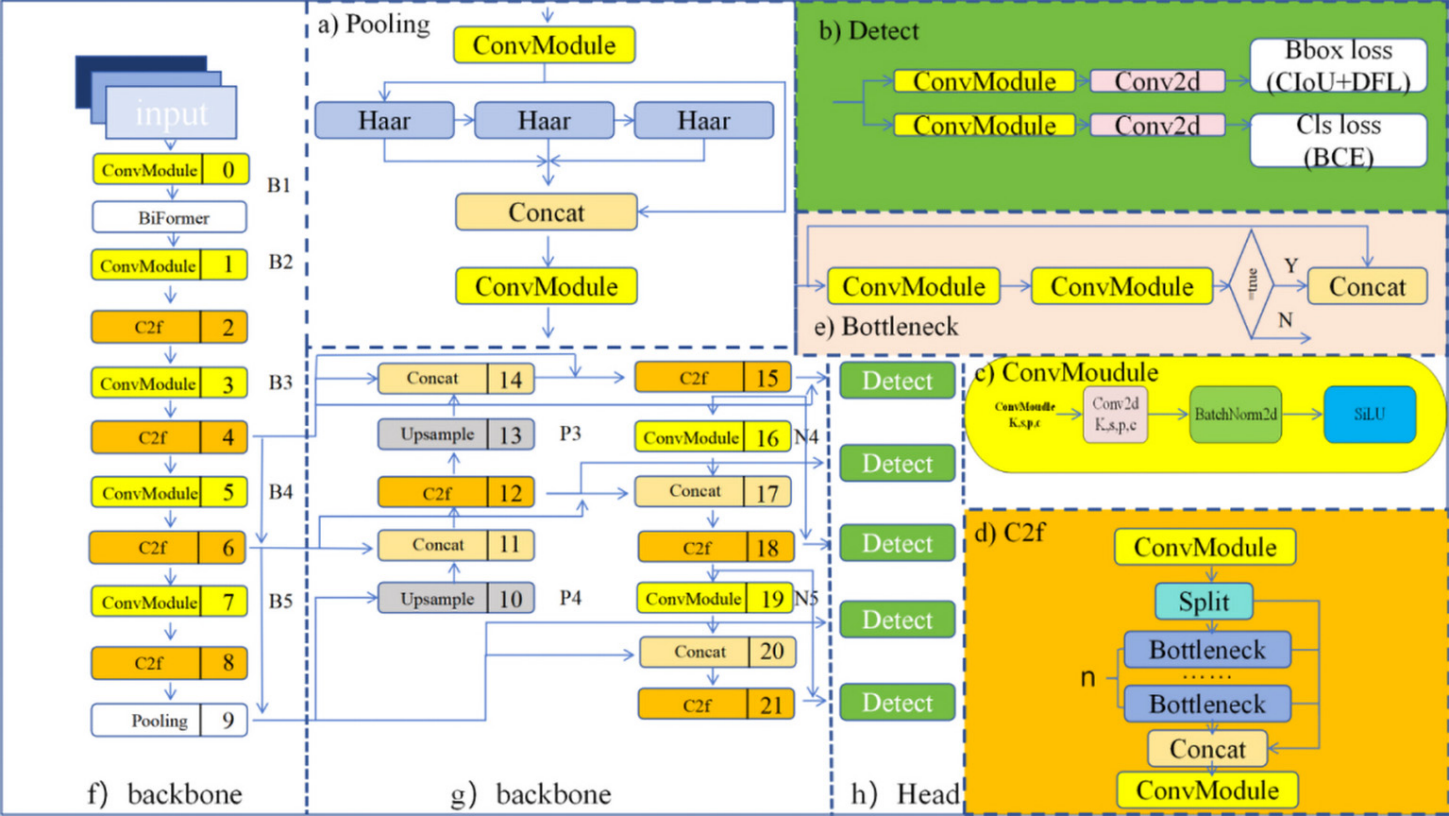
YOLOv8 architecture diagram. **(A)** Pooling. **(B)** Detect. **(C)** ConvMoudule. **(D)** C2f. **(E)** Bottleneck. **(F)** backbone. **(G)** backbone. **(H)** Head.

The model in this paper adopts an improved CSPDarknet53 as the backbone network (Wang et al., 2023) for YOLOv8. It conducts down sampling on input features five times, resulting in five different scales of features, denoted as B1 to B5. The structure of the backbone network is illustrated in **Figure 2F**. The CSP (Cross Stage Partial) module in the original backbone network of previous versions is replaced by the C2f module. The structure of the C2f module is shown in **Figure 2D**, where ‘n’ represents the number of bottlenecks. The C2f module adopts gradient parallel connections, enriching the information flow of the feature extraction network while maintaining a lightweight design. The ConvModule module conducts convolutional operations on input information, followed by batch normalization, and then utilizes the SiLU activation function to obtain the output result, as shown in **Figure 2C**. The backbone network concludes by utilizing an improved down sampling module to pool input feature maps into fixed-size adaptive-sized outputs. Compared to the original Spatial Pyramid Pooling (SPPF) structure, the new connection layers can retain more feature information, as shown in **Figure 2A**.

Inspired by PANet, the original YOLOv8 incorporates a PAN-FPN structure at the neck (Wang et al., 2023). Compared to the neck structures of YOLOv5 and YOLOv7 models, YOLOv8 removes the convolutional operation after up sampling in the PAN structure, as shown in **Figure 2E** achieving model lightweighting while maintaining the original performance. YOLOv8 adopts a top-down and bottom-up network structure to integrate semantic information from deep and shallow features. However, this fusion is superficial. To address this, we designed a new feature fusion network based on the PAN-FPN architecture. Through the analysis of tea leaf defect images, it was determined that spatial positional information of features is not necessary in practical applications. Therefore, part of the feature information flow can be trimmed to reduce computational costs. Simultaneously, feature fusion is achieved by merging different nodes of the same feature layer, retaining more features of tea pests and diseases without increasing computational costs.

The detection part of YOLOv8 adopts a decoupled head structure, as shown in **Figure 2B**. This structure employs two independent branches for object classification and bounding box regression prediction, each using different loss functions. For the classification task, binary cross-entropy loss (BCELoss) is used. For the bounding box regression task, Distribution Focal Loss (DFL) and Complete Intersection over Union (CIoU) are employed. This detection structure improves detection accuracy and accelerates model convergence. YOLOv8 is an anchor-free detection model, which simplifies the specification of positive and negative samples. It also utilizes the Task-Aligned Assigner to dynamically assign samples, enhancing the detection accuracy and robustness of the model.

### 2.3 Bi Former

To focus the detection model on tea leaf defects and diseases while reducing attention on other regions, we introduce a dynamic sparse attention mechanism called Bi Former (Zhu et al., 2023) into the backbone network of the model. Bi Former utilizes adaptive querying to filter out the least relevant key-value pairs in the coarse-grained regions of the input feature map. It then efficiently identifies the key-value pairs with higher relevance and performs attention computation on them. This significantly reduces computational and storage costs, enhancing the model’s ability to perceive the input content. YOLOv8 is a convolutional neural network (CNN) model. The essence of a CNN is local processing, which limits its ability to capture relationships between global features. Compared to traditional CNN models, transformers use an attention mechanism to capture the relationships between different pieces of data, providing a global receptive field. An effective attention mechanism can build robust and powerful data-driven models, making them more flexible when handling complex, large-scale data.

**Figure 3:**
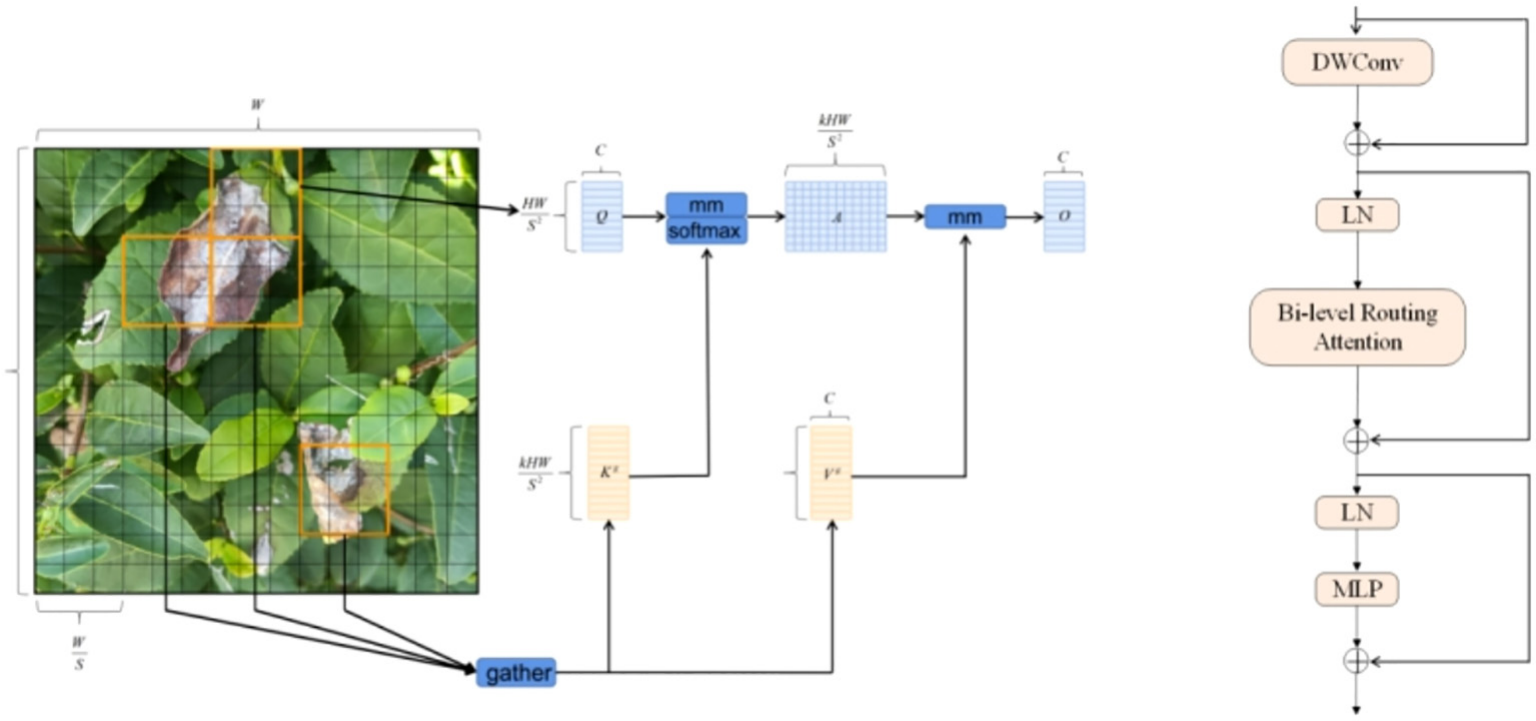
Principle and operational diagram of BI FORMER.

The Bi Former module is designed based on a dual-stage routing attention mechanism, as shown in **Figure 3**. In this block, DW Conv represents depth wise separable convolution, which reduces the number of parameters and the computational load of the model. LN stands for layer normalization, which accelerates training and improves the model’s generalization ability. MLP, or multilayer perceptron, further processes and adjusts attention weights, enhancing the model’s focus on different features. The addition symbol in **Figure 3** represents the concatenation of two feature vectors.

The introduction of the Bi Former block into the backbone network in this paper serves two purposes. First, Bi Former considers the limited computational power and storage resources of mobile platforms. Second, the dynamic attention mechanism within this block enhances the model’s focus on crucial target information, thereby optimizing the model’s detection performance. To fully leverage the efficient attention mechanism of this block, we added the Bi Former block between the model backbone networks B1 and B2.

### 2.4 Down sampling

Down sampling can aggregate local information, expand the receptive field, and reduce computational costs. Conventional down sampling operations mainly involve max-pooling and stride convolution. However, pooling operations on local regions can lead to the loss of important spatial information, which is detrimental to accurate detection. To address this, we introduce down sampling operations based on the Haar (Xu et al., 2023) wavelet.

**Figure 4:**
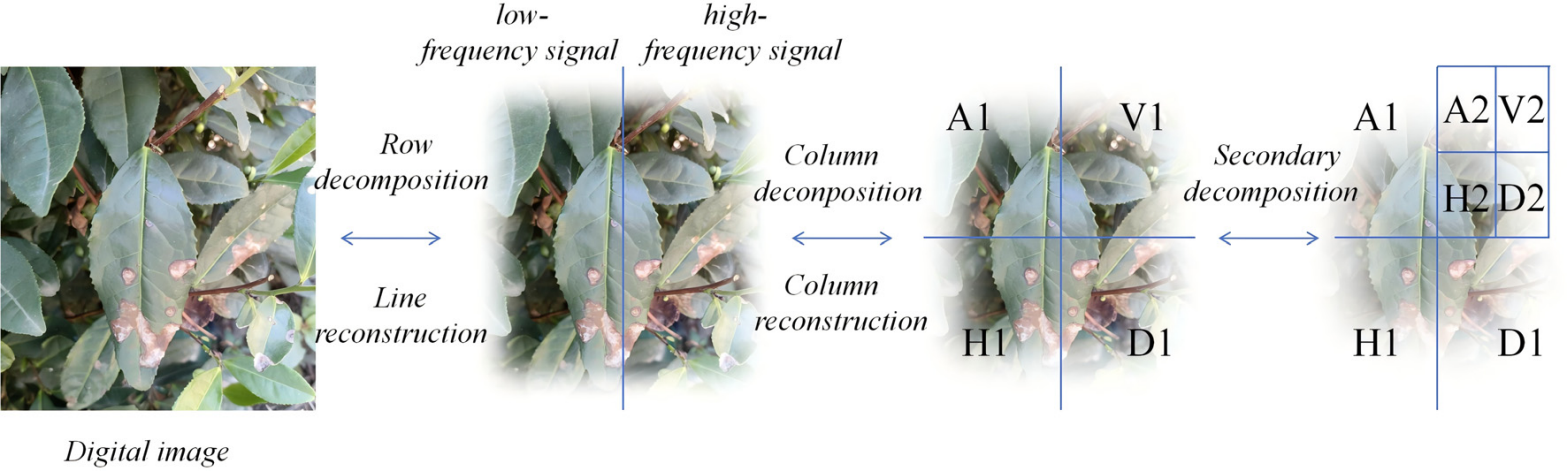
The operational process of the Haar wavelet.

The core idea of the new down sampling operation is to use Haar wavelet transformation to reduce the spatial resolution of feature maps while preserving more information. This approach enhances the ability of semantic segmentation and reduces information uncertainty. For 2D image Haar decomposition, it can be seen as performing 1D Haar decomposition separately on all columns and all rows. Depending on the order of decomposing rows and columns, two different decomposition methods can be generated. The specific process is shown in **Figure 4**.

**Figure 5:**
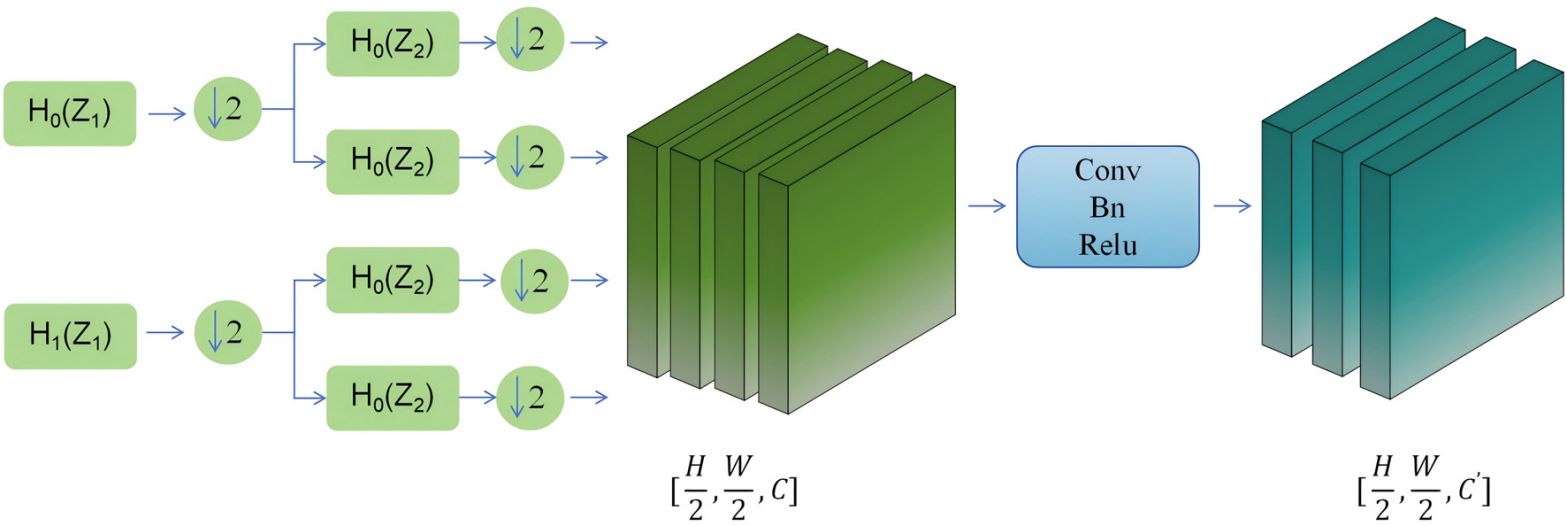
Downsampling Module.

From **Figure 5** it can be seen that the module first preprocesses the input information flow in the horizontal and vertical directions by performing averaging and differencing operations on the information flow separately. Then, down sampling is performed. Next, the processed information flow undergoes diagonal direction processing, where it is averaged and differenced to obtain diagonal subbands. Each of these subbands is then down sampled. This process iteratively repeats for each subband.

Finally, an inverse transformation is applied to each subband to reconstruct the original image. These steps constitute the lossless feature encoding module primarily based on the Haar wavelet transform. Subsequently, the output information flow undergoes convolution, normalization, and activation function processing to reduce the number of channels.

### 2.5 Feature fusion

YOLOv8 itself uses a simplified FPN-PANet in its neck to perform feature fusion, reducing the loss of information. The core idea of FPN is to construct a feature pyramid at different levels of the image to capture objects at different scales: by up sampling the deep feature maps to match the size of the shallow feature maps (Gong et al., 2021), and then performing an addition operation. PAN, on the other hand, employs a cascaded operation, which can retain more detailed information, thereby improving detection accuracy (Wang et al., 2019).

However, the above operations have two drawbacks: first, they do not focus on features at the same level; second, the merging process can introduce delays, leading to suboptimal merging effects. Considering that in tea plantation inspections using drones, multiple photos are taken of the same area, the edge features of a single photo are not our primary concern. At the same time, when the drone takes photos, it is approximately 50 cm away from the top of the tea trees. Each photo contains a large number of tea leaves, which implies there are many instances of defects and diseases.

To address these issues, our approach focuses on refining the feature fusion process to enhance the detection of tea leaf defects and diseases in such scenarios. By prioritizing crucial target features and optimizing the merging process, we aim to achieve more accurate and efficient detection results.

**Figure 6:**
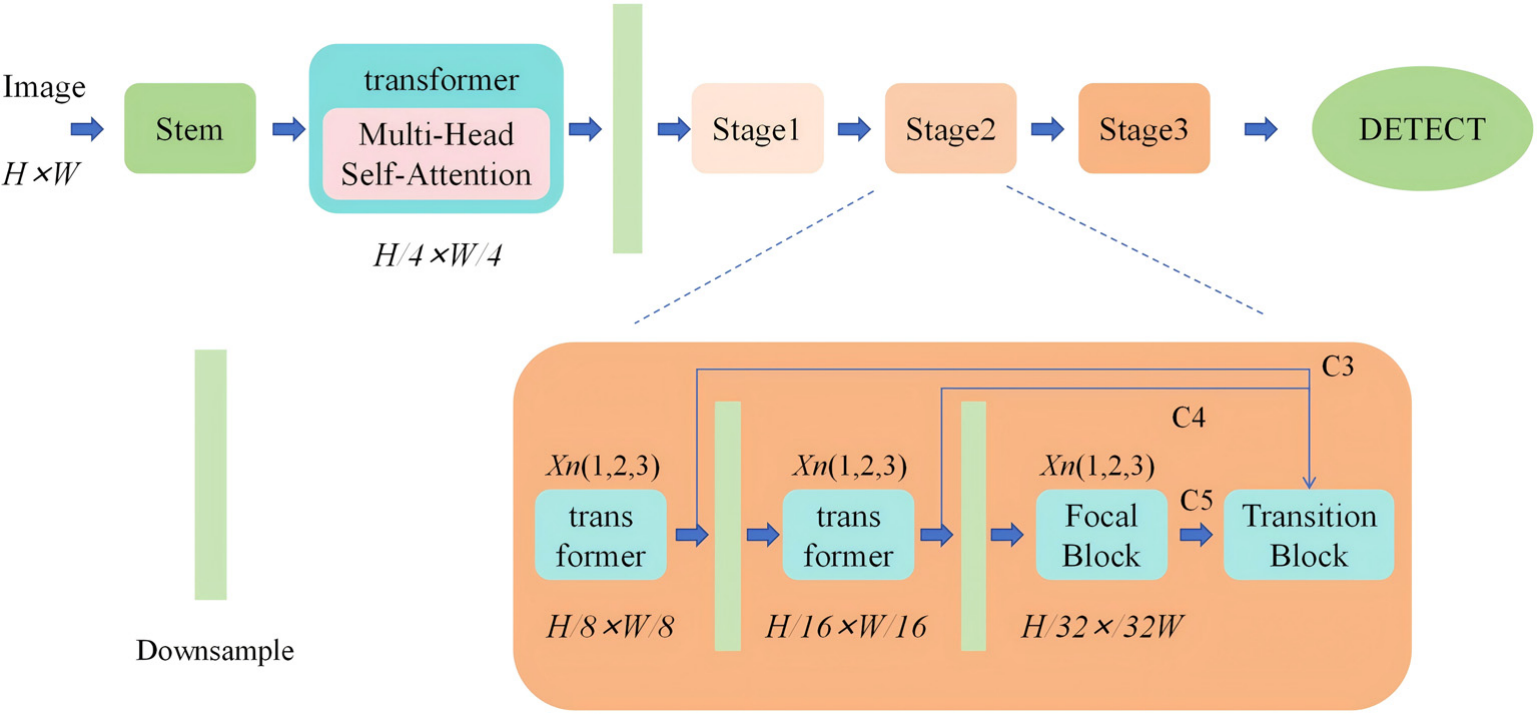
Arithmetic unit structure diagram.

Based on the limited receptive field of CNN networks, they can only localize regions with distinctiveness. As shown in **Figure 6**, Therefore, the first step is to use a multi-head attention mechanism to segment the image into patches with distinctive features. Since deep features reflect specific information about objects and require global context, a transformer encoder is used to process deep features to enhance object detection performance.

**Figure 7:**
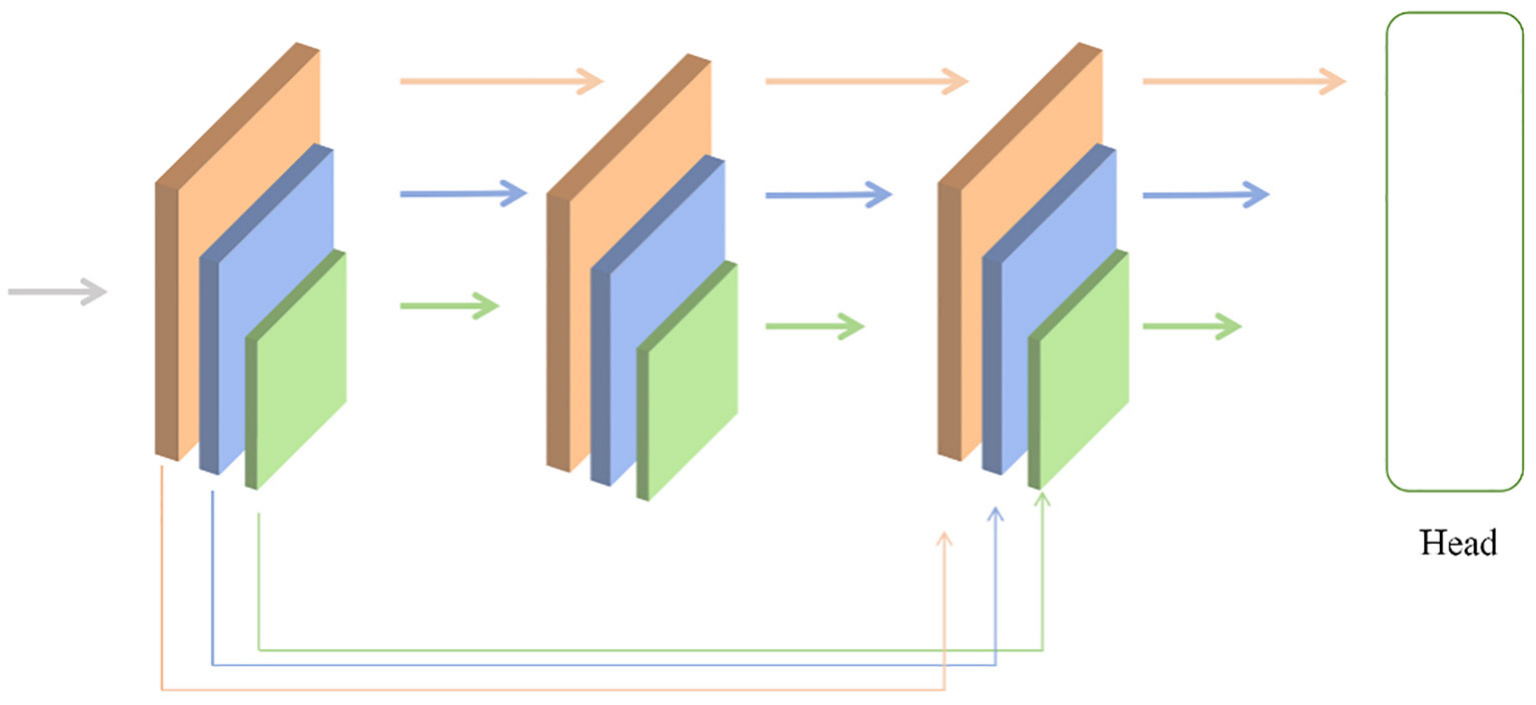
The running diagram in the model.

Next, under the condition of unchanged computational resources, allocating more parameters for feature fusion can be achieved by intuitively reducing the backbone layers and expanding the fusion modules. From **Figure 7**, To achieve this, the backbone network is decomposed into several smaller cascaded stages, generating richer scale features. Each stage consists of a sub-backbone and a transformation module.

Performing skip connections on the same feature layer helps preserve more feature information. The Transition block utilizes 1x1 convolutions to align the channel numbers in the sampling points and uses bilinear interpolation to align the spatial sizes of features. The Focal Block, on the other hand, enlarges the convolutional kernel to expand the receptive field, thereby acquiring more feature information.

By implementing these modifications, the model can better handle complex scenes with multiple instances of tea leaf defects and diseases, improving detection accuracy and robustness.

The contributions of features from images with different resolutions are unequal, hence an additional weight is introduced for learning. Building upon Unbounded Fusion, normalization of weights is conducted to constrain the value range of each weight. Unbounded Fusion refers to integrating features from different resolutions without explicit boundaries.

## 3 Results and discussion

### 3.1 Experimental facilities

To verify the positive impact of each module on the model, YOLOv8n was used as the baseline model, and ablation experiments were conducted separately on the BiFormer module, Haar module, and feature fusion module. In order to ensure the accuracy of the experimental results, the parameter settings in each individual module are the same.

At the same time, in order to ensure that the pre trained weight structure and the target model structure are the same in the experiment, all three experimental groups will undergo weight pre training before the formal experiment, and the weight pre training dataset will use the dataset from Chapter 2, which will not cause overfitting.

### 3.2 Ablation experiment

#### 3.2.1 The attention mechanism comparative experiment

To verify the superiority of introducing Bi Former, we conducted comparative experiments using Bi Former and some mainstream attention mechanisms on the YOLOv8n baseline model while keeping other training conditions consistent. The experimental results, as shown in **Table 1** indicate that when BiFormer is incorporated into the backbone network of the model, it achieves the best detection performance. Furthermore, the model with the attention module incorporated shows a 16.5% increase in mAP50 compared to when the attention module is not introduced.

**Table 1:** Detection results of different attention mechanism.

Metrics	Precision/%	Recall%	mAP0.5/%	mAP0.5:0.95/%
Nothing	80.4	64.8	72.2	47.7
SE	81.1	63.0	70.1	49.2
CBAM	81.0	71.1	70.2	49.5
ECA	80.3	68.5	69.9	48.3
ContextAggregation	84.9	75.2	83.3	61.5
BIFORMER	**89.8**	**82.3**	**88.7**	**65.9**

Bold indicates the optimal value of the current indicator.

For achieving optimal performance after adding the Bi Former block, this paper conducted the following comparative experiments. We used YOLOv8n as the baseline model and added Bi Former blocks at different layers of the backbone network. The results are shown in **Table 2** From the experimental results, it can be observed that adding the Bi Former block to deeper layers of the network leads to higher detection performance, but also increases computational complexity. Adding Bi Former to layers B4-B5 increased the computational load by 9.5 times, yet the improvements in various metrics were less than 3%. In order to balance detection performance and computational requirements, this paper added the Bi Former block to layers B1-B2.

**Table 2:** Detection results of different depths of Bi Former module.

Model	Precision/%	Recall%	mAP0.5/%	mAP0.5:0.95/%	FLOPS
B1-B2	89.8	82.3	88.7	65.9	17.6G
B2-B3	89.1	81.4	86.5	62.2	35.2G
B3-B4	90.2	83.3	89.1	67.4	78.9G
B4-B5	91.1	83.6	90.0	68.6	168.2G

In the experimental results, we can see that the total amount calculated by BiFormer varies greatly at different depths, but the difference in results is not significant. This is because the module runs in four stages, each of which reduces the resolution of the input image while increasing the number of channels *C*. The total calculation amount is shown in the following formula:


(1) $$FLOPs=3HWC^{2}+3Ck^{\frac{2}{3}}{\left(2HW\right)}^{\frac{4}{3}}$$

Where *k* is the number of regions to participate in.

The number of channels in feature maps with different layers will increase with the increase of layers.

BIformer will divide the input sequence into two parts, performing self-attention calculation and cross attention calculation respectively. The former captures the internal dependencies of the sequence, while the latter captures the dependencies between sequences. Although they perform better when placed at a deeper level, their principle is to filter out key value pairs that are irrelevant to the query at a coarse-grained level, and adaptively focus on the most relevant key value pairs at a fine-grained level; placing it into a deeper network can provide it with more detailed information, but shallower networks can also provide the vast majority of key information, so its performance growth is not significant.

#### 3.2.2 Haar wavelet experiment

In convolutional neural networks, pooling layers are used to reduce the spatial size of data, decrease computational complexity, while retaining important features. Commonly used pooling methods include: max pooling, average pooling, and adaptive average pooling.

**Figure 8:**
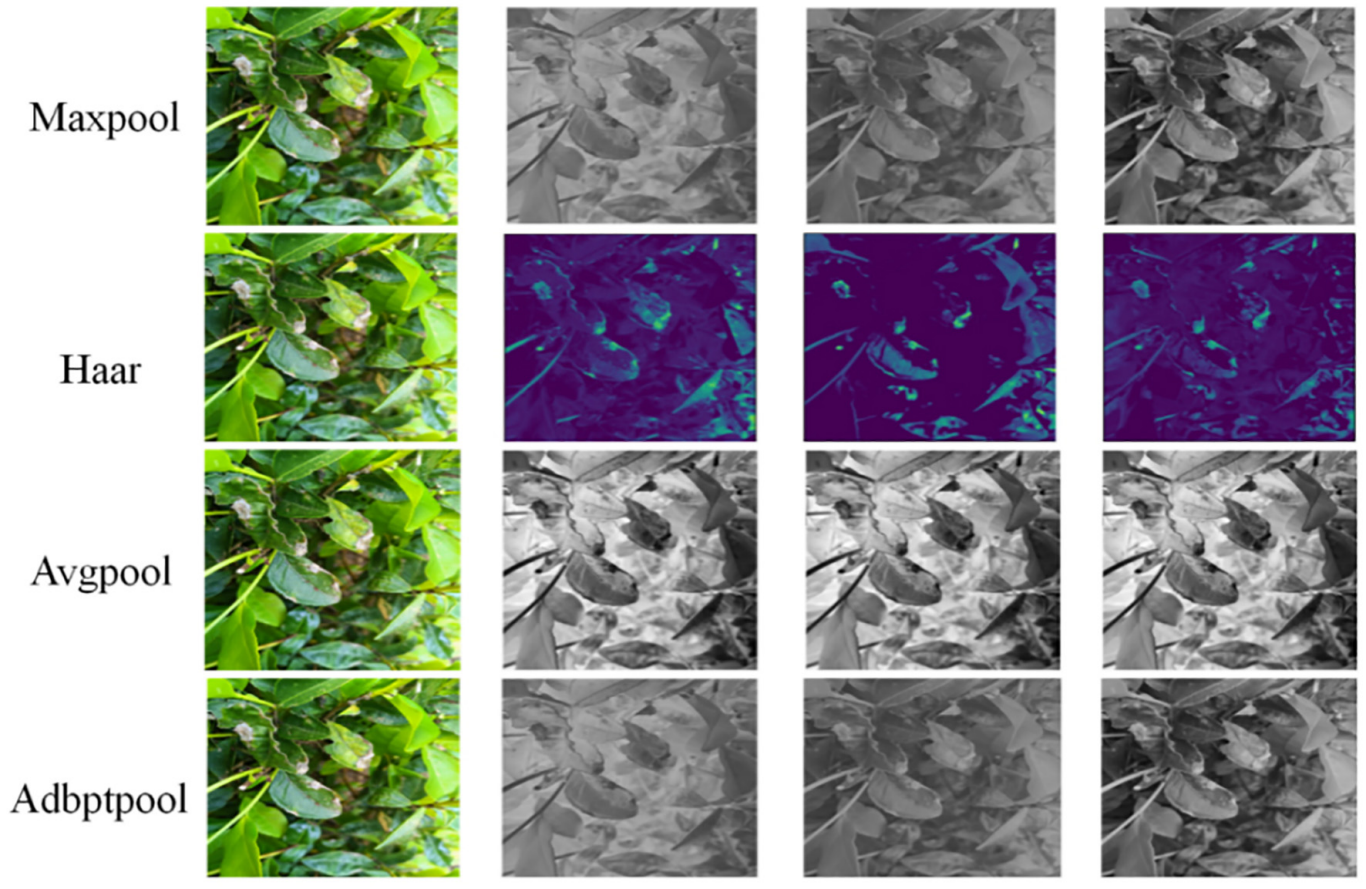
Comparison of different pooling methods.

Pooling convolutional layers can easily lose feature data and spatial location information, affecting detection performance. In the baseline model of YOLOv8n, spatial pyramid pooling is used when transitioning from the backbone network to deeper layers. The fundamental unit of spatial pyramid pooling is max pooling. Although it improves upon the drawbacks of max pooling, it still cannot entirely avoid the loss of feature information. The paper introduces a down sampling module based on Haar wavelet functions and compares it with common pooling methods. The results are shown in **Figure 8**. When inputting the same image, it’s evident that Haar wavelet-based pooling can preserve feature and spatial information to a greater extent. From **Table 3** we can see that Haar has higher confidence and mAP than its peers, but its recall rate is not as good as Adbptpool. This is because Adbptpool adaptively calculates weights, which increases its computational load. Maxpool is the most commonly used method, with relatively balanced performance but not as high accuracy as Haar. In summary, we ultimately used Haar as the downsampling module.

**Table 3:** Comparison of different pooling methods.

Metrics	Precision/%	Recall%	mAP0.5/%	mAP0.5:0.95/%
Maxpool	66.8	55.1	63.2	37.2
Haar	**70.2**	58.2	**69.5**	**44.6**
Avgpool	60.5	50.6	57.1	31.4
Adbptpool	62.3	**58.9**	61.2	36.1

Bold indicates the optimal value of the current indicator.

#### 3.2.3 Feature fusion network

In YOLOv8, the feature fusion network in the backbone is FPN-PANet. To validate the improved feature fusion network proposed in this paper, comparative experiments were conducted using v8n as the baseline model. Several mainstream feature fusion structures were also compared. The results are shown in **Table 4**. From the table, we can observe that compared to the baseline modelFPN, as the earliest proposed pyramid network, is the foundation for subsequent multi-scale network design. Its disadvantages are twofold. Firstly, it only adopts a top-down path, resulting in insufficient low-level information; secondly, it lacks dynamic weights, leading to underutilization of some important features. PANet introduced bidirectional paths, increasing the complexity of feature fusion, but its performance was not as expected in complex backgrounds. The NAS-FPN architecture is optimized for specific tasks and datasets, with high search costs and complex structures. BiFPN can learn weight dependencies, but it is prone to getting stuck in local optima, resulting in limited performance improvement. The model proposed in this article considers the characteristics of tea disease detection tasks and takes into account practical application situations, partially introducing bidirectional paths and weight dependencies.

**Table 4:** Detection results of different feature fusion networks.

Model	Precision/%	Recall%	mAP0.5/%	mAP0.5:0.95/%	FLOPS
FPN	78.9	58.1	63.1	37.2	**7.9G**
PANet	79.2	58.3	62.4	38.4	10.4G
NAS-FPN	78.5	57.6	62.6	37.6	9.3G
FPN-PANet	81.0	65.8	72.2	48.1	15.2G
BIFPN	83.7	**76.3**	83.1	62.0	22.7G
Ours	**86.4**	75.9	**86.3**	**63.4**	12.1G

Bold indicates the optimal value of the current indicator.

From the table, we can observe that compared to the baseline model, our feature fusion structure exhibits better detection accuracy, with a 19.5% increase in mAP0.5, while the computational complexity decreases by 20.4%. Therefore, it can be concluded that our structure preserves more feature information during feature fusion with minimal computational overhead.

### 3.3 Comparative experiment

To demonstrate the superiority and effectiveness of the proposed improved algorithm, we conducted comparative experiments. First, we compared various models in the YOLO series: YOLOv3 (Redmon and Farhadi, 2018) nd its lightweight version YOLOv3-tiny (Adarsh et al., 2020); YOLOv4 (Bochkovskiy et al., 2020) with the novel backbone network CSPDarknet53; YOLOv5n (Xue et al., 2023), which improves accuracy using mosaic data augmentation; and YOLOv9s (Wang et al., 2024a), which introduces new structures based on YOLOv7. Also, we compared the tea detection model developed by YOLO-Tea (Xue et al., 2023), Hossain et al. (2018) and TSBA-YOLO (Lin et al., 2023), which can now be applied to the prevention and control of tea diseases and pests.

From **Table 5** we can analogize the advantages and disadvantages of the model proposed in the above paper. Compared to models such as YOLOv3 and YOLOv5, the later proposed YOLOv8 and v9 have better performance, with mAP reaching over 70%. However, this is still not an ideal accuracy rate. Because these four models are only a framework and do not specifically detect the characteristics of tea pests, diseases, and defects in images. However, YOLOv10b and YOLOv11n are improvements based on YOLOv8 and YOLOv9, still retaining similar shortcomings. Therefore, subsequent research mainly focuses on targeted optimization of this drawback, such as the attention mechanism and feature fusion module proposed in this paper, which take into account the characteristics of tea damage and the features captured during drone inspections. After targeted optimization, our model achieved a precision of 92.2% and an mAP of 94.5%, far exceeding similar models.

**Table 5:** Test results of different models.

Model	Precision/%	Recall%	mAP0.5/%	mAP0.5:0.95/%
YOLOv3	49.5	40.3	37.2	18.6
YOLOv3-tiny	39.2	31.7	29.5	17.9
YOLOv4	57.8	45.4	48.4	25.5
YOLOv5n	71.0	64.8	65.7	37.6
YOLOv8n	80.2	69.7	72.2	47.3
YOLOv9s	79.8	75.0	75.2	45.0
YOLOv10b	81.2	77.8	83.9	68.2
YOLOv11n	86.2	80.4	87.3	70.1
Hossain S	72.3	74.2	68.6	43.1
TSBA-YOLO	67.6	81.5	71.5	51.2
YOLO-Tea	85.1	85.7	86.5	64.7
Ours	**92.2**	**87.1**	**94.5**	**71.4**

Bold indicates the optimal value of the current indicator.

## 4 Conclusions

Due to the texture, shape, and color characteristics of tea leaves, accurately detecting defects and pest damage is challenging. The small size of the leaves, in particular, renders existing models insufficient for our research needs. Therefore, we have enhanced the YOLOV8n model in various ways to improve its detection capabilities for tea leaf defects and diseases.

(Chen et al., 2024b) proposed a new ViTNet model, which mainly detects smile pest and disease features by introducing self-attention mechanism and global feature extraction. Secondly, the EMA PANet model was introduced to improve the multi-scale information acquisition ability (Chen et al., 2024a) proposed using transfer learning and freezing core strategies to improve timely detection ability (Li et al., 2024) proposed embedding the CA attention mechanism into MobileNetV2 and proposed a multi branch parallel strategy to extract features, which can adapt to different diseases. And use AutoML for Model Compression (AMC) to compress the computational load (Zhou et al., 2024) proposes to use the GS DeepLabV3 network, only Chen paid attention to the attention mechanism, which can effectively reduce computational complexity and improve accuracy. However, the adaptive attention mechanism used by Chen calculates global features, which requires a large amount of computation; the EMA PANet model is a feature fusion network based on PANet, which improves performance by adding fusion paths, but this can lead to difficulty in training and slow convergence. Transfer learning and freezing core strategies can lead to poor generalization performance of the model and neglect of underlying features. The multi branch parallel strategy proposed by Li for feature extraction is a great method.

Our model combines their strengths and discards their weaknesses. Firstly, because YOLOV8 struggles to focus on small targets such as disease defects, we employed the Bi Former attention mechanism to direct the model’s attention towards these areas. Bi Former filters out irrelevant feature information at the upper layers of the network, retaining only a portion of the regions. Within these regions, it then utilizes token-to-token attention for higher precision. The DWconv reduces computational load, and the MLP adjusts the attention weights accordingly (Chen et al., 2024a).

Secondly, the baseline model’s max pyramid pooling employs a max pooling module. As shown in **Figure 7**, the effective information retained by max pooling is not highly sensitive to tea leaf defects and diseases. However, pooling operations using the Haar function can preserve more feature information. The Haar function can retain essential feature information to the greatest extent when transmission channel performance is suboptimal, then reconstruct the image for the next layer of computation. During this process, feature maps computed using the Haar function are able to preserve critical information to the maximum extent.

Finally, the new feature fusion network decomposes the backbone network into sub-backbone networks with distinct features under the transform framework. This leverages the parallel processing advantages of GPUs, thereby accelerating computation speed. When processing single features, the model often exhibits better performance. Additionally, by summing the feature maps of the same layer, more feature information can be retained without increasing computational load.

**Figure 9:**
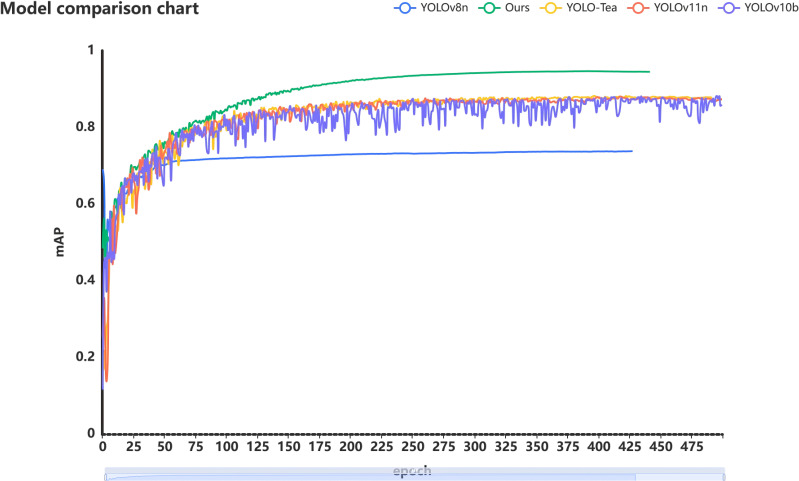
Comparison of several excellent model results.

Through a series of improvements, we ultimately developed the BHC-YOLO model for detecting tea leaf defects and diseases. As shown in **Figure 9**, the BHC model outperforms other tea leaf detection models available on the market. Notably, the dataset considers the impact of weather factors on practicality, and the algorithm enhances the original images, thereby increasing the model’s generalization capability.

However, there are still shortcomings and areas for improvement in this model. Firstly, the computational complexity is still relatively high, which requires a certain level of power consumption for portable artificial intelligence chipsets and is not easy to carry. In the subsequent work, we will prune the entire model to further reduce computational complexity. Secondly, there is a high demand for photo quality, and once in a low light environment, the accuracy will suddenly decrease; the recognition rate of sporadic tea pests and diseases is low, and there is still room for improvement.

## Data Availability

The raw data supporting the conclusions of this article will be made available by the authors, without undue reservation.
